# Non-destructive Analysis of the Mechanical Properties of 3D-Printed Materials

**DOI:** 10.1007/s10921-022-00854-5

**Published:** 2022-02-18

**Authors:** R. Domingo-Roca, L. Asciak, J. F. C. Windmill, H. Mulvana, J. C. Jackson-Camargo

**Affiliations:** 1 Department of Biomedical Engineering, The Wolfson Centre, University of Strathclyde, 106 Rottenrow Street, Glasgow, G4 0NW UK; 2 Department of Electronic & Electrical Engineering, University of Strathclyde, Glasgow, UK

**Keywords:** 3D-printing, Digital-light processing, Non-destructive, Mechanical properties, Euler–Bernoulli, Laser-scanning vibrometry

## Abstract

The determination of the mechanical properties of materials is predominantly undertaken using destructive approaches. Such approaches are based on well-established mathematical formulations where a physical property of the material is measured as a function of an input under controlled conditions provided by some machine, such as load–displacement curves in indentation tests and stress–strain plots in tensile testing. The main disadvantage of these methods is that they involve destruction of samples as they are usually tested to failure to determine the properties of interest. This means that large sample sizes are required to obtain statistical certainty, a condition that, depending on the material, may mean the process is both time consuming and expensive. In addition, for rapid prototyping and small-batch manufacturing of polymers, these techniques may be inappropriate either due to excessive cost or high polymer composition variability between batches. In this paper we discuss how the Euler–Bernoulli beam theory can be exploited for experimental, non-destructive assessment of the mechanical properties of three different 3D-printed materials: a plastic, an elastomer, and a hydrogel. We demonstrate applicability of the approach for materials, which vary by several orders of magnitude of Young’s moduli, by measuring the resonance frequencies of appended rectangular cantilevers using laser Doppler vibrometry. The results indicate that experimental determination of the resonance frequency can be used to accurately determine the exact elastic modulus of any given 3D-printed component. We compare the obtained results with those obtained by tensile testing for comparison and validation.

## Introduction

3D printers are becoming extremely popular in scientific research as a result of the wide range of potential applications they are suited to, from soft robotics to biomedical research [1–4]. Since the early 1980s a range of 3D-printing approaches have been developed to rapidly produce structures using different materials with consequently differing properties [5], each with their own set of advantages and limitations. There are numerous examples in the literature of how different 3D-printing methods, namely fused deposition modelling (FDM), stereolithography (STL), or digital light processing (DLP), can produce materials with contrasting mechanical properties—from soft to hard plastics, alloys, hydrogels, and elastomers—that can be applied in several areas of scientific research [6–10]. The 3D-printing revolution took a step further forward with the development of functional materials (namely conductive, piezoelectric, magnetic, thermo-responsive, cell-compatible) that can be used to produce a wide variety of sensors and actuators at macro-, meso-, and micro-scales. In a matter of a few years, sensors that required complex manufacturing processes and long production times could be fabricated in a single print, simply by combining different materials with different properties.

Irrespective of the method used to 3D-print, there is a common uniting factor—the final parts are made of amorphous materials. This happens as a consequence of the high temperatures applied in FDM (typically within a range of 200–230 °C, such that the thermoplastic filaments can be melted and re-solidified) or the use of polymer-based, photo-responsive resins in SLA and DLP approaches (which are solidified upon exposure to light). 3D-printing remains an active area of research, often involving long print times and the use of expensive resins as the drive to optimise performance and material properties. Hence, determination of the mechanical properties of the resulting materials, irrespective of the final application, ultimate geometry, or functionality, is essential to assess applicability for any given application.

Mechanical testing is synonymous with movement: determining the mechanical properties of materials is linked to their deformation. All techniques—tensile testing, indentation, rheology—require deformation (in some cases breakage) of the sample, a feature that may be highly inconvenient for certain areas of research. There is another question that arises: How are the mechanical properties of engineered materials (such as layered composites, for instance) calculated? Many applications require multiple materials to be produced in specific arrangements such as layers, reinforced structures, or particle suspensions. Mathematical models exist in the literature to allow mechanical properties of these new materials to be estimated as a function of the matrix and filler densities and concentrations [11], but they still require individual testing of the different components for accurate results. These theoretical formulations are based on assumptions regarding the homogeneity of any filler distribution, uniformity of particle size, and isotropic properties. However, since 3D-printed materials are not typically isotropic, the use of such assumptions will give rise to errors. Methods such as indentation are limited to specific materials. Mechanical properties are calculated based on the projected area of the indenting tip employed, leading to variability as a function of tip geometry (spherical, flat, Berkovich, Vickers, Knoop, etc.) [12] and the need for large datasets and averaging to obtain accuracy. Furthermore, the indents must be separated a given distance (which depends on the material and the size of the tip) to minimise the influence of neighbouring indents, while the indentation penetration depth should not exceed 10% of the sample thickness. As a result, indentation becomes a less precise technique when dealing with inhomogeneous suspensions and layered materials. While tensile and compression testing seem to provide a better solution to this problem, they assess material properties in one particular direction at a time and the resulting mechanical properties are directional and usually orthogonal. For 3D-printed materials, the mechanical properties will also be dependent on layer orientation and exposure time in addition to any post-curing and post-processing of parts, leading to high variability and thus meaning that large sample sizes are required for accuracy. In addition, tensile and compression testing require samples with specific dimensions. Usually, these dimensions are much larger than a typical 3D-print (especially in SLA and DLP approaches), which may require the sample to be re-oriented within the active 3D-printing area to produce a sample for test. This will often incur long print times (> 24 h) and mean that materials can only be tested and mechanical properties derived in one orientation. While moulds have been developed to address this issue (and to eliminate the presence of layers in the 3D-printed part), this is not an optimum solution, especially for light-responsive materials. Their characteristic light penetration depth, which determines the polymerisation kinetics (ruled by the Beer–Lambert law), implies solidification of the top layers while the bottom ones may not reach gel point. This limitation results in longer exposure times, therefore resulting in inhomogeneous crosslinking densities within the material.

We demonstrate the use of the Euler–Bernoulli beam theory to experimentally determine the elastic modulus of 3D-printed materials, including plastics, elastomers, and hydrogels, which range from GPa to a few kPa. By investigating several materials we demonstrate accurate measurement of the Young’s modulus in all of them, such that the method that we propose can be universally applied. We show how 3D-printing rectangular cantilevers (of user-defined dimensions) can provide an estimation of the Young’s modulus of the sample by experimentally measuring their resonance frequency and applying the equations of the Euler–Bernoulli beam theory. To do so, we used a laser Doppler vibrometer to measure the resonance frequencies of sets of cantilevers of varying lengths (from 3 to 7 mm) and obtain their surface displacement. The mechanical properties of 3D-printed parts are highly dependent on a large set of parameters, including exposure time, layer thickness, filler concentration (if used), and layer orientation. The method described here provides the tools to determine, non-destructively, the mechanical properties of a 3D-printed part simply by embedding a set of cantilevers within the sample. The method offers a set of advantages: (i) only one sample needs to be 3D-printed, therefore optimising the production process and time, (ii) complex calculations for particle distribution, layer orientation, and other factors influencing the mechanical properties of the sample are not required, as the very same sample is measured in a non-destructive manner, and (iii) given that the technique is non-destructive, the same sample can be repeatedly tested to provide large, reliable data sets. We compare the results obtained using this method to those obtained by tensile testing, a well-established method highly used for determining the mechanical properties of materials.

## Materials and Methods

### The Euler–Bernoulli Beam Theory

Several beam theories exist, each with a set of assumptions that influence accuracy and applicability. One of the simplest methods is the well-known Euler–Bernoulli beam theory, which assumes that the cross-section of the beam is infinitely rigid in its own plane, therefore leading to zero deformations in that very same plane, supporting the assumption that the deformations in that plane are zero [13]. As a consequence of this assumption, the in-plane displacement field can be described by two rigid body translations and one rigid body rotation. Two additional assumptions can be made to account for out-of-plane displacement: (i) the cross-section is assumed to remain planar after deformation, and (ii) the cross-section is assumed to remain normal to the deformed axis of the beam during deformation [13].

Hence, the Euler–Bernoulli equation describes the deflection (*z*) on a beam as a consequence of an applied load (*p*) according to a second order differential equation (Eq. (1)).

1 $$\frac{{d}^{2}}{d{x}^{2}}\left(EI\frac{{d}^{2}z}{d{x}^{2}}\right)=p$$

where *E* is the elastic modulus of the material, and *I* is the second moment of inertia (or area moment of inertia) of the beam’s cross-section. Under oscillatory loads we must consider both kinetic and potential energy terms [13], which lead to Eq. (2).

2 $$\frac{{\partial }^{2}}{\partial {x}^{2}}\left(EI\frac{{\partial }^{2}z}{\partial {x}^{2}}\right)=-\mu \frac{{\partial }^{2}z}{\partial {t}^{2}}+p(x)$$

where $\mu$ is the mass per unit length. In the free vibration regime (*i.e.*, in the absence of load), the general solution to Eq. (2) is given by the Fourier decomposition of the sum of the harmonic vibrations, introducing the angular frequency *ω*. This leads to general solutions of Eq. (2) that are a combination of sine, cosine, hyperbolic sine, and hyperbolic cosine, all of them as a function of ${\beta =\left(\mu {\omega }^{2}/EI\right)}^{1/4}$.

When fixing one of the edges of the beam (i.e., cantilevered beam), the boundary conditions change, leading to zero vertical displacement and velocity at *x* = 0. These, together with the additional boundary conditions due to the free edge of the cantilever, imply that non-trivial solutions to Eq. (2) are found to exist only if $\mathrm{cosh}\left({\beta }_{n}L\right)+\mathrm{cos}\left({\beta }_{n}L\right)+1=0$, leading to natural angular frequencies of vibration given by Eq. (3) (where *β*_*n*_ roots can be determined analytically).

3 $${\omega }_{n}={\beta }_{n}^{2}\sqrt{\frac{EI}{\mu }}\Rightarrow {\omega }_{1}=\frac{3.5161}{{L}^{2}}\sqrt{\frac{EI}{\mu }}$$

Introducing the analytical expression of *I* into Eq. (3) for a rectangular beam and solving for *E*, it is straight-forward to obtain Eq. (4).

4 $$E=\frac{48\rho {\pi }^{2}}{3.5161}{\left(\frac{{f}_{1}{L}^{2}}{h}\right)}^{2}$$

where *h* is the thickness of the beam, $\rho$ is the density of the material, and *f*_*1*_ the natural frequency of vibration of the beam.

The maths underpinning the processes described are well-established and detailed derivation of Eqs. (2)–(4) can be found in the literature [13]. It must also be considered that unsupported beams will present different boundary conditions, while different geometries (such as circular beams, for instance) will lead to alterations in the moment of inertia, directly influencing Eq. (4).

### 3D-Printing and Hydrogel Preparation

Three 3D-printing material formulations were studied: a hard plastic (Orange Tough®, PRUSA, Prague, Czechia), an elastomer (Formlabs Flexible 50A®, Formlabs, Massacusetts, US), and a custom hydrogel. Two different 3D printers were used (PRUSA SL1, and ASIGA Pico2HD27UV) to 3D-print the samples. The hydrogel was developed using bisphenol-A ethoxylate dimethacrylate (BEMA, 1.7 kDa, Sigma Aldrich) in de-ionized water (20 wt%) with lithium phenyl-2,4,6-trimethylbenzoylphosphinate (LAP, Sigma Aldrich) and tartrazine (Sigma Aldrich) at varying concentrations. The mixture was stirred for 30 min prior to 3D-printing. The commercial materials were used as received.

**Fig. 1:**
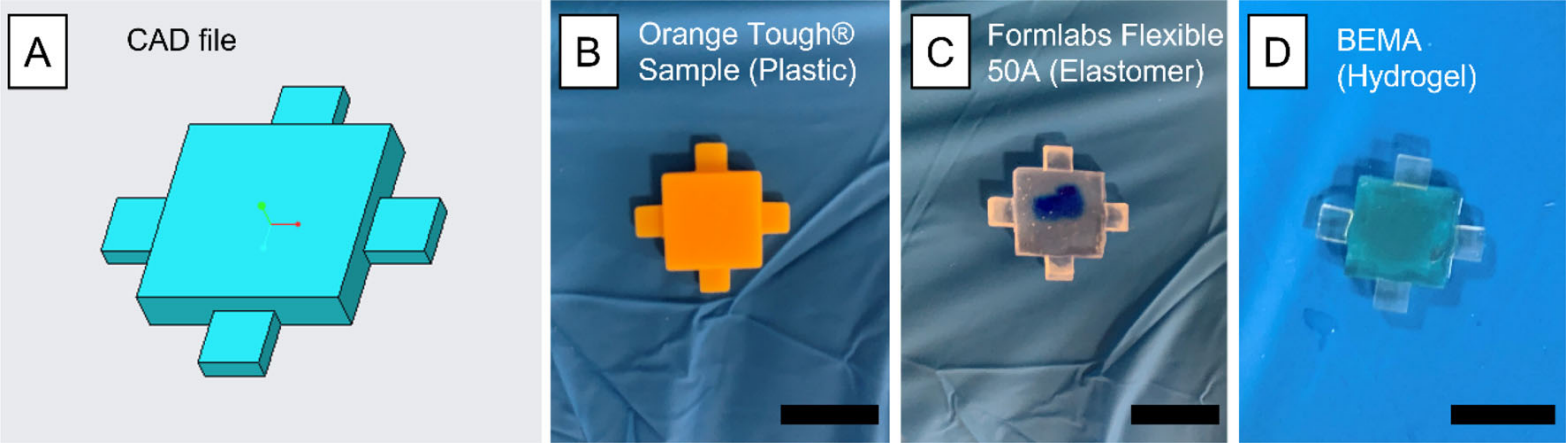
**A** CAD generated to 3D-print the samples for mechanical testing. **B**–**D** 3D-printed samples for non-destructive mechanical testing using Orange Tough®, Formlabs® elastomer, and BEMA hydrogel (20 wt%), respectively. Orange Tough® was 3D-printed using a PRUSA SL1, while the Formlabs® elastomer and the hydrogel were 3D-printed using an ASIGA® UV 3D printer. These samples correspond to cantilever lengths of 6 mm. Samples with cantilever lengths of 3 mm, 4 mm, 5 mm, and 7 mm were 3D-printed using all materials. **D** Includes the 3D-printed base layer supporting the 3D-printing process of the hydrogel, required to enhance adhesion between the hydrogel and the build block. Scale bars, 10 mm

Samples containing cantilevers of different lengths (from 3 to 7 mm in steps of 1 mm) were 3D-printed using Orange Tough®, Formlabs Flexible 50A®, and BEMA hydrogel (20 wt% in dH_2_O). Figure 1 shows one of the computer-aided design (CAD) files used to produce the samples (a different CAD file was used for each cantilever length) and the corresponding 3D-printed parts in plastic, elastomer, and hydrogel (Fig. 1B–D). After 3D-printing, the samples were thoroughly washed in isopropyl alcohol and sonicated for 10 min. Recent studies have revealed that the use of solvents like IPA to post-process 3D-printed polymers induces an extra degree of stress to the samples [14], leading to deformation of their geometry and, most importantly, modification of their mechanical properties.

After washing, samples were placed in a UV chamber for 10 min to ensure full polymerisation of the 3D-printed parts.

### Tensile Testing

To confirm the validity of this approach, tensile testing (Instron, Electropuls E10000) was performed to determine the elastic modulus of the different samples using a conventional, well-established method. The machine was equipped with a 1kN biaxial dynamic load cell. The tensile testing experiments used a 5-s hold period followed by a ramp stage at a crosshead speed of (a) 1 mm/min until specimen failure for the hydrogels, and (b) 10 mm/min for the elastomer. The 3D-printed samples had a total width of 12 mm (gauge width 4 mm), a uniform thickness of 3 mm, and a total length of 50 mm (gauge length, 20 mm). Displacement and load data were recorded in order to calculate both strain and stress, which were plotted to determine the elastic modulus of the materials from the elastic region of the stress–strain graphs. Orange Tough® was validated versus the elastic modulus values supplied by the manufacturer.

### Laser Doppler Vibrometry

**Fig. 2:**
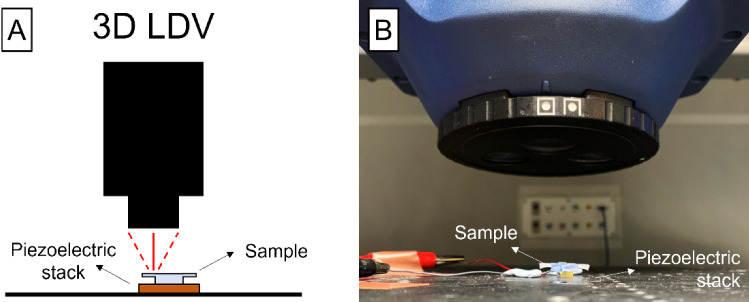
**A** A schematic of the laser Doppler vibrometer (LDV) head, the piezoelectric stack, and the sample. The LDV scans the surface of the sample by moving the base stage to the corresponding scanning location. **B** Picture of the experimental set up

The natural frequencies of the 3D-printed samples were assessed using a 3D laser Doppler vibrometer (3D LDV) system with an MSA-100-3D scanning head (Polytec, Waldbrom, Germany). The response of the 3D-printed beams was studied under stimulation using a piezoelectric stack (TA0505D024W, Thorlabs) driven with a periodic chirp from 20 Hz to 5 kHz at peak-to-peak voltage of 1 V and 0.5 V offset. The piezoelectric stack was driven using the 3D LDV internal signal generator. Point measurements were used for the calculations and full scans of the beam’s surface deflection were performed for better illustration (Polytec System Analyzer). Figure 2 shows the experimental set up that we used to evaluate the resonance frequencies of the cantilevers.

Laser Doppler vibrometry (LDV) is a powerful tool able to detect sub-nanometre motion in a contactless manner in all three dimensions of space. LDVs are based on a Mach–Zehnder interferometer. Briefly, the laser beam is focussed onto the vibrating surface of the sample and scattered back. This backscattered signal is Doppler-shifted arising as a consequence of the velocity of motion of the sample. Using this approach, we are able to scan the full surface of the cantilever (at a user-defined point resolution, in our case 2 points per mm provided a good representation of surface deflection) and see the mode shape at a given frequency and determine the characteristic frequencies of the cantilevers. Using these frequencies, we can replace the value in Eq. (4) and determine what is the corresponding Young’s modulus of the sample of interest.

## Results

### Tensile Testing

**Fig. 3:**
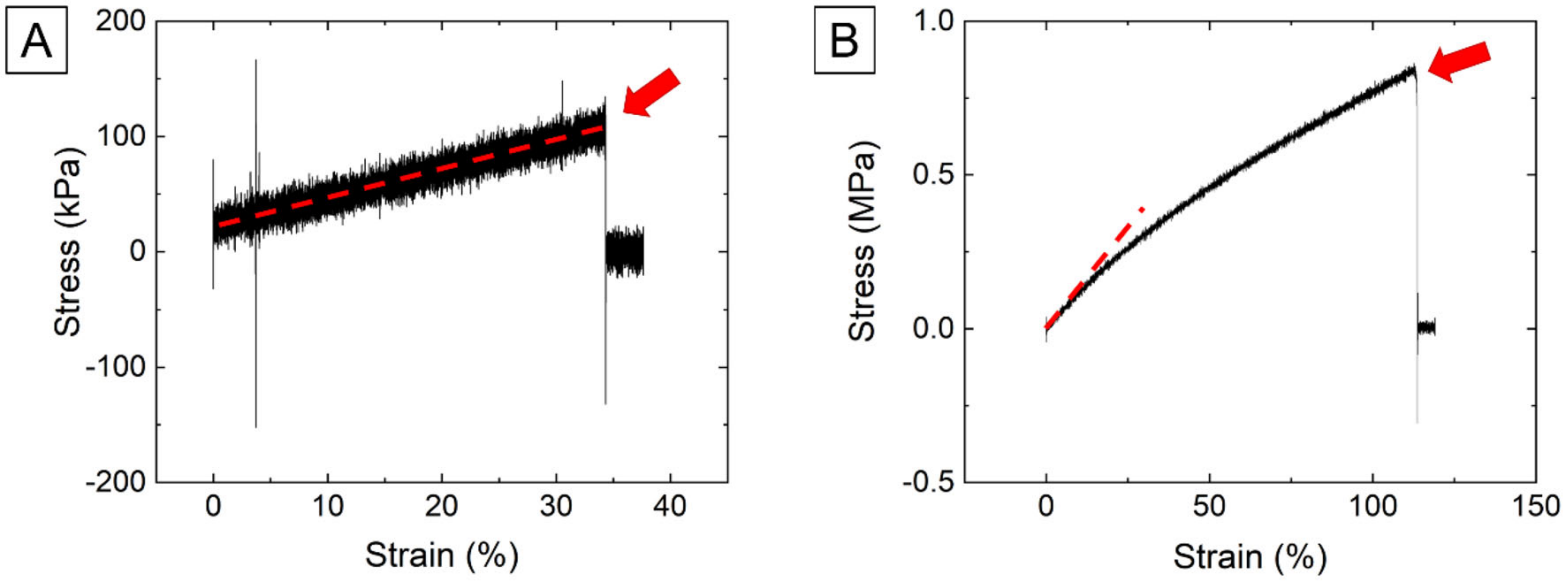
Stress–strain curves obtained from tensile testing experiments. **A** Hydrogel, and **B** elastomer. The arrows indicate the point where the samples broke. Dashed lines indicate the elastic region used to calculate the elastic modulus

Young’s modulus (*E*) of the elastomer and the hydrogel were determined (*N* = 5) from tensile testing analyses (Instron Electropuls, E10000). Two representative stress–strain curves of these materials are shown in Fig. 3, with arrows indicating breakage of the samples.

Tensile testing provided values of tensile elastic modulus (± s.d.) of 215.25 ± 79.42 kPa and 1.132 ± 0.039 MPa for the hydrogel and elastomer, respectively. While most of the hydrogel samples revealed a linear behaviour, two of them were observed to deviate from this linearity close to breakage (around 22% applied strain). The elastic modulus calculated from tensile testing for the Formlabs® elastomer matches that reported by the manufacturer [15], and matching typical elastic modulus values of shore 50A elastomers within the range 1.34 and 1.70 MPa [16].

### Laser Doppler Vibrometry and Application of the Euler–Bernoulli Beam Theory

**Fig. 4:**
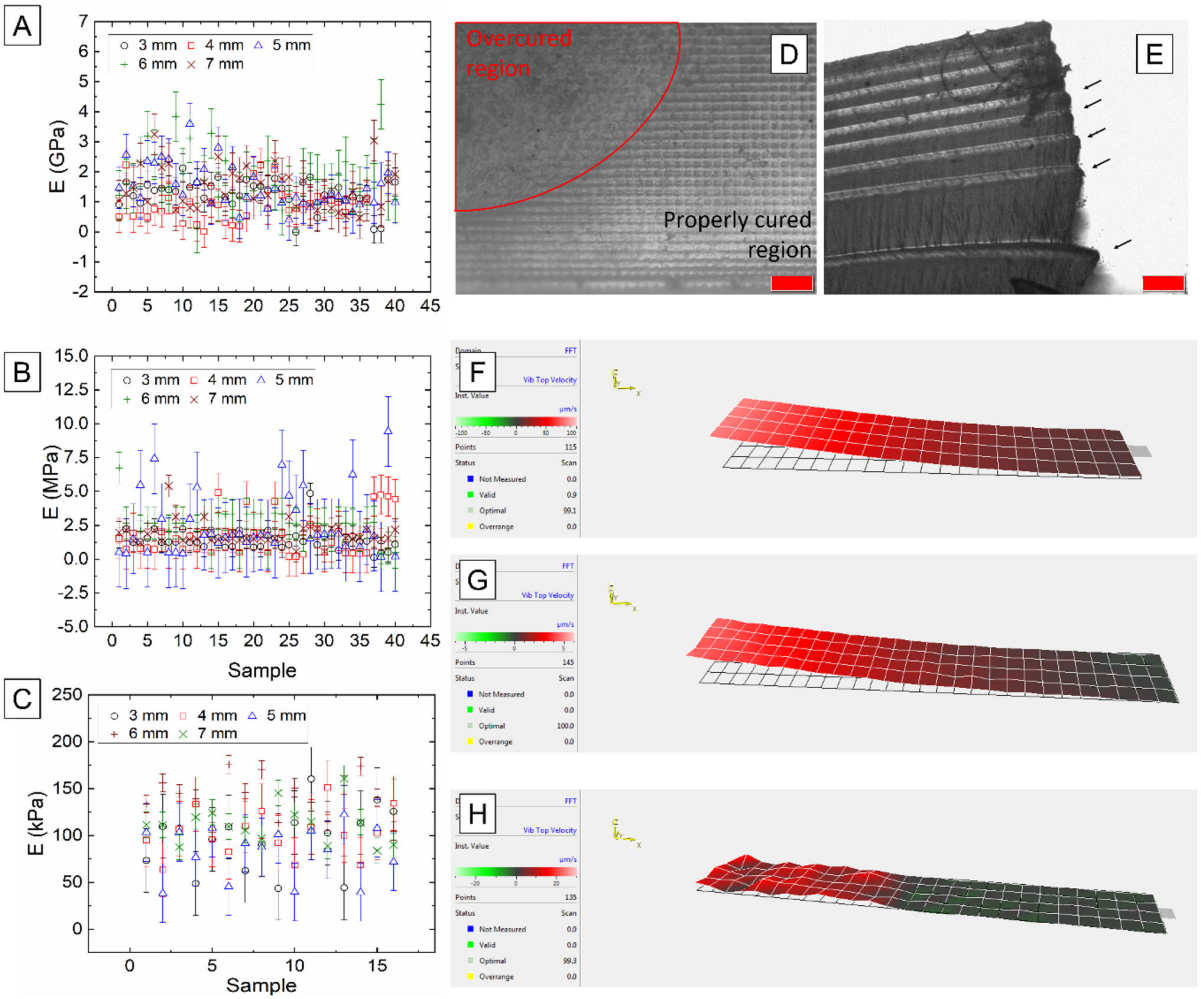
**A**–**C** show the elastic modulus of Orange Tough®, Formlabs® 50A elastomer (*N* = 120), and hydrogel (*N* = 28). **D** Shows a micrograph of an overcured region of material in Orange Tough® (scale bar, 200 µm). **E** Shows a micrograph of the detachment of the 3D-printed layers in Formlabs® 50A elastomer, attributed to the post processing required after sample 3D-printing (scale bar, 200 µm). **F**–**H** Show the first natural resonance frequency of Orange Tough®, Formlabs® 50A elastomer, and hydrogel, acquired using 3D laser Doppler vibrometry, and corresponding to frequencies of 3.055 kHz, 207.90 Hz, and 33.15 Hz, respectively, for cantilever lengths of 7 mm. Dark areas indicate regions of zero displacement

The first resonance frequencies of the 3D-printed rectangular cantilevers were determined by scanning their surface deflection while undergoing excitation. The obtained frequency values were used to calculate the elastic moduli of all the 3D-printed materials using Eq. (4). The obtained results are shown in Fig. 4A–C, where the Young’s moduli corresponding to the different cantilever lengths are plotted as a function of sample number. We measured less hydrogel samples due to dehydration; as the experiments are performed in air, hydrogels continuously lose water, which requires constant rehydration between measurements to ensure results reliability. Variance in the data points can be observed in Fig. 4A–C. This is attributed to regions of Orange Tough® that have been overcured (*i.e.*, that have received extra UV exposure during 3D-printing, Fig. 4D) or not uniformly post-processed. The variance observed in the elastomer data is attributed to this very same post-processing process, which induced detachment of the 3D-printed layers composing the sample (Fig. 4E, where the arrows indicate the individual layers that have been detached from each other). Variance in hydrogel samples are attributed to the natural dehydration process that hydrogels undergo. This dehydration process, which becomes critical after 5 min in our case, is a consequence of the time required to undertake surface scans. Within this study scans took between 3 and 10 min, depending on cantilever length. After 5 min, the samples started to dehydrate due to the porous nature of hydrogels, and this mass loss directly influences their resonance frequencies and, therefore, their mechanical properties (according to Eqs. (3) and (4)).

**Fig. 5:**
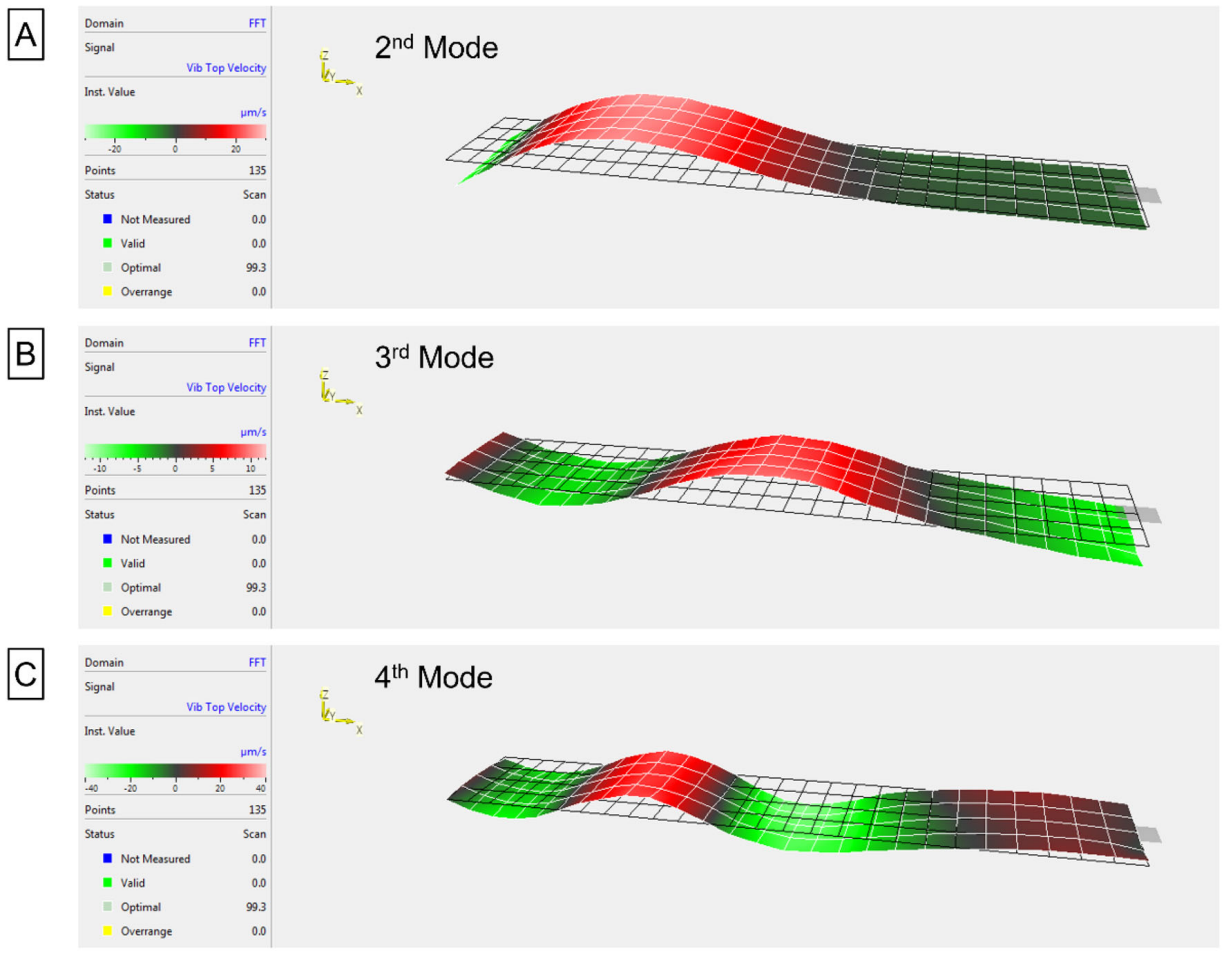
**A**–**C** Show the surface scans of the second, third, and fourth (respectively) natural resonance frequencies of 7 mm long rectangular cantilevers. These surface scans correspond to the hydrogel sample, which was constantly re-hydrated (by wetting the sample with water using a 1 mL pipette) during the scanning process to ensure data reliability. The frequencies of these modal shapes correspond, from **A** to **C**, to 216.3 Hz, 456.3 Hz, and 896.3 Hz

The first natural frequencies used to determine the elastic moduli are shown in Fig. 4F–H. 3D laser Doppler vibrometry surface scans confirmed the expected modal behaviour of all the 3D-printed samples. The obtained frequencies led to average elastic moduli values (± s.e.) of 1.35 ± 0.10 GPa, 2.05 ± 0.21 MPa, and 105.44 ± 0.10 kPa for Orange Tough®, elastomer, and hydrogel, respectively. These values are in good agreement with those measured during tensile testing and those reported by the manufacturers, with values of 1.14–1.25 GPa for Orange Tough® and 1.34–1.70 MPa for the elastomer [16, 17]. Full surface scans using frequency sweeps (from 10 Hz to 20 kHz) were performed to confirm the presence of higher modes (Fig. 5) and corroborate that the obtained results are not machine or sample artifacts.

## Discussion

The laser Doppler vibrometer has allowed us to measure the surface vibration of cantilevers of 3, 4, 5 6, and 7 mm in length. We have experimentally observed the presence of the first, second, third, and fourth mode (Fig. 4F–H and Fig. 5) for all cantilever lengths, hence demonstrating that accurate determination of the characteristic frequencies can be achieved following this method. By obtaining these frequencies, we have been able to successfully replace their values in Eq. (4) and determine, in a non-destructive manner, the Young’s modulus of samples that go from hundreds of kPa to a few GPa (Fig. 4A–C).

Our results show good agreement with the properties reported by the manufacturers and more commonly used techniques such as tensile testing are obtained (here employed as a confirmatory technique), there are a set of factors that must be considered. Firstly, post-processing of the parts—solvent washing plays a key role in the final geometry of the 3D-printed samples (as a consequence of inducing extra levels of stress, especially when using IPA or acetone), therefore influencing mechanical properties (Fig. 4D, E). The nature of any post-processing required is dependent on material type and as a result the influence on the structure’s mechanical properties will vary. This underlines the need to establish methods to accurately characterise mechanical properties of structures once printed and in situ rather than relying on complex mathematical models that make specific sets of hypotheses and that may not necessarily match the real experimental conditions.

Secondly, the viscoelastic and shear-thickening behaviour of elastomers and hydrogels means that the resulting mechanical properties measured will depend on the testing protocol employed and will vary with parameters such as loading rate. This means that where rheology, indentation, and tensile testing are conducted under different (or non-equivalent) conditions they will provide different mechanical properties. Thirdly, the porous nature of hydrogels means that they dehydrate over time. This has significant consequences when measuring their mechanical properties and means that hydrogels must remain in their fully swollen state for both repeatability and in order to compare with results against those found in the literature. Finally, it must not be forgotten, that the Euler–Bernoulli beam theory implies a set of assumptions that may lead to slight variability. Our results demonstrate that this method can achieve an excellent match to the mechanical property values reported in the literature and by the manufacturers of the materials we investigated without the need for destructive testing.

## Conclusions

We have demonstrated that the Euler–Bernoulli beam theory can be used to determine, in a non-destructive manner, the elastic modulus of 3D-printed samples. We further demonstrate that the Euler–Bernoulli beam theory can be applied to a wide range of materials with a large range of elastic moduli, from a few kPa to several GPa. By scanning the surface vibration of cantilevers of known geometries, it is possible to determine their resonant frequencies and simply substitute them in Eq. (4) to determine the Young’s modulus of the sample of interest (as all the other parameters are either known or directly measurable). While the use of hydrogels requires special attention of the sample (constant rehydration), the method has shown to be valid for this type of materials. This method provides a big advantage over destructive measuring methods such as giving the user the possibility to directly measure the sample of interest by simply adding a little cantilever in the part. This feature enables direct measurement of the Young’s modulus of very specific 3D-printed materials (layered materials, particle suspensions, layer orientation, etc.) without relying on complex mathematical models.

We propose that this measurement technique will allow manufacturers to customise their parts by including cantilevers allowing accurate determination of elastic modulus while also minimising production time, material used, and avoiding sample breakage.

## Data Availability

The data required to reproduce the work presented in this paper can be accessed through PURE Portal, the University of Strathclyde’s research information portal.
